# The State of Open-Heart Surgery in Nigeria: Challenges, Opportunities, and the Way Forward

**DOI:** 10.7759/cureus.100692

**Published:** 2026-01-03

**Authors:** Obinna Ikegwuonu, Chibuike F Obi, Ayo-Oladapo Kolawole, Marthins Akhigbe, Victor O Elekwa

**Affiliations:** 1 Orthopedics and Traumatology, Surgery Interest Group of Africa, Lagos, NGA; 2 Medicine, Fairfield General Hospital, Northern Care Alliance NHS Foundation Trust, Manchester, GBR; 3 Orthopedics, National Orthopaedic Hospital, Igbobi, Lagos, NGA; 4 Urology, Lagos State University Teaching Hospital, Lagos, NGA; 5 General Surgery, Ebonyi State University, Abakaliki, NGA

**Keywords:** challenges and barriers, nigeria, open cardiac surgery, open-heart surgery, opportunities

## Abstract

Nigeria is facing an increasing burden of cardiovascular diseases, creating an urgent need for sustainable open-heart surgery (OHS) services. Despite decades having passed since the first OHS, the country’s progress has been inconsistent, with most centers still operating below capacity. In this paper, we reviewed the existing literature to identify the major challenges facing OHS in Nigeria and to highlight potential opportunities and future directions. Data were collected from articles and reports sourced from PubMed and Google Scholar. The search included keywords such as “challenges”, “difficulties”, “barriers”, “open heart surgery”, “cardiac surgery”, “Nigeria”, and “Federal Republic of Nigeria”. Articles that discussed barriers, opportunities, and recommendations for improving OHS services were included. The major challenges identified include inadequate infrastructure and equipment, a shortage of skilled personnel, limited funding and affordability, weak systemic policy, low case volume, and poor incentives and retention. Reasonable opportunities lie in fostering international and diaspora collaborations, increasing private sector involvement and partnerships, and leveraging the epidemiological need. Future directions include strengthening cardiac capacity through sustainable financing mechanisms, workforce development, and integration of OHS into national health policies and insurance schemes. Nigeria’s OHS services remain fragile but not without promise. By addressing systemic gaps and capitalizing on emerging opportunities, the country can transition from episodic, externally driven cardiac missions to reliable, self-sustaining national OHS programs.

## Introduction and background

Open-heart surgery (OHS) stands among the most intricate and resource-intensive interventions in modern medicine, offering lifesaving treatment for a wide range of congenital and acquired cardiac conditions. While the procedure has become routine in many high-income countries, its availability in Nigeria remains limited, with progress unfolding slowly over the past five decades. The country recorded its first OHS on 1 February 1974 at the University of Nigeria Teaching Hospital (UNTH), Enugu, a milestone that demonstrated local feasibility but did not translate into sustained national capacity [1]. Subsequent decades saw intermittent activity across a handful of centers, punctuated by periods of dormancy tied to funding, staffing, and equipment constraints [1,2].

Despite a growing burden of operable cardiac disease, ranging from rheumatic valvular lesions to congenital defects, Nigeria’s OHS services remain uneven and often mission-dependent. Recurring barriers include shortages of trained personnel, unreliable supply chains for consumables and prostheses, inconsistent power supply and critical care infrastructure, and high patient costs. Multi-year reports from UNTH, Lagos State University Teaching Hospital (LASUTH), and other centers describe stop-start programs, visiting teams, and backlogs of patients who either wait or travel abroad [1,3].

Financing remains a major constraint. Despite the Abuja Declaration’s 15% benchmark, Nigeria allocates less than 5% of its federal budget to health, resulting in persistently low investment. Consequently, private and out-of-pocket spending, estimated at roughly three-quarters of total health expenditure, dominates the system. For high-cost, technology-intensive services such as OHS, this financing structure renders care largely inaccessible for many households [4-6].

Health insurance has not yet filled the gap. Although Nigeria reformed its scheme into the National Health Insurance Authority (NHIA), population coverage remains in the single digits, with multiple 2024-2025 analyses noting <10% enrollment and wide state-level variation. Limited pooling, fragmented purchaser-provider arrangements, and weak benefits for tertiary care constrain the capacity to underwrite complex cardiac surgery [7,8].

This review aims to synthesize the existing challenges and emerging opportunities in OHS in Nigeria, while critically assessing the strategic steps required to advance the field from episodic activities to a more sustainable national cardiac surgery capacity.

## Review

Current state of OHS in Nigeria

OHS in Nigeria is currently undertaken in a handful of public and private centers across key states, including Lagos (LASUTH), Enugu (UNTH), and, more recently, Abuja (Federal Medical Centre), among others. For example, LASUTH initiated its OHS program in 2004 with mission support and gradually transitioned toward greater indigenous team participation [9]. In Enugu, the National Cardiothoracic Centre was revived through repeated foreign visits from 2013 to 2019, completing over 266 operations during that period [10]. The newer Abuja program began OHS in 2022 under a hybrid mission-local model; in its first six months, seven cases were performed, including one entirely by local staff [11].

Across the country, the Nigeria Open-Heart Surgery Registry publishes operational data per center (e.g., procedure counts, adult versus pediatric case mix, and local versus visiting team contributions) and shows that many centers still rely partially on visiting teams [12]. Despite these advances, access remains extremely limited: approximately 100-130 heart procedures are completed annually nationwide, amounting to coverage of less than 0.00006% of the population [13].

Workforce constraints remain acute. There are very few trained local cardiothoracic surgeons, perfusionists, and intensive care staff, and much of the training still occurs abroad or through mission teams [1,11,14]. Geographic inequities persist, as many regions have no nearby centers; consequently, patients must travel long distances or are unable to access surgery at all [1,9,11]. The registry helps highlight these gaps and guide planning for expansion [12].

Methods

Our preliminary search revealed that the available qualitative studies varied considerably in scope, design, and reporting, making them unsuitable for formal systematic synthesis or qualitative metasynthesis, as recommended by Dixon-Woods et al. and Sandelowski et al. [15,16]. Therefore, we conducted a narrative review [17]. Data for this review were collected from articles identified through keyword searches on PubMed and Google Scholar, without restricting the search to a specific time period. The keywords searched included “challenges”, “difficulties”, “barriers”, “open heart surgery”, “cardiac surgery”, “Nigeria”, and “Federal Republic of Nigeria”. Using these keywords, we developed a search strategy, which is shown in Table 1.

**Table 1:** Search strategy

Database	Search terms	Number of articles
PubMed	challenges OR difficulties OR barriers OR obstacles OR limitations AND “open cardiac surgery” OR “cardiac operation” OR “heart operation” OR “cardiothoracic operation” OR “coronary artery bypass grafting” OR “valve surgery*” OR “cardiac bypass surgery*” OR “heart transplant surgery*” AND Nigeria OR Nigerian OR “Federal Republic of Nigeria”	9
Google Scholar	challenges AND “open heart surgery” OR “cardiac surgery” OR “heart surgery” AND Nigeria	999

We included only published articles relevant to OHS in Nigeria that discussed challenges and barriers, opportunities, and potential ways forward. Commentaries, editorials, and expert opinion pieces providing insights into challenges and solutions were also considered. Studies were excluded if they lacked Nigeria-specific data or focused solely on pan-African or general cardiothoracic surgery. Conference abstracts, inaccessible full texts, and letters, commentaries, or expert opinions without substantial discussion of challenges, opportunities, or next steps were also excluded.

**Figure 1:**
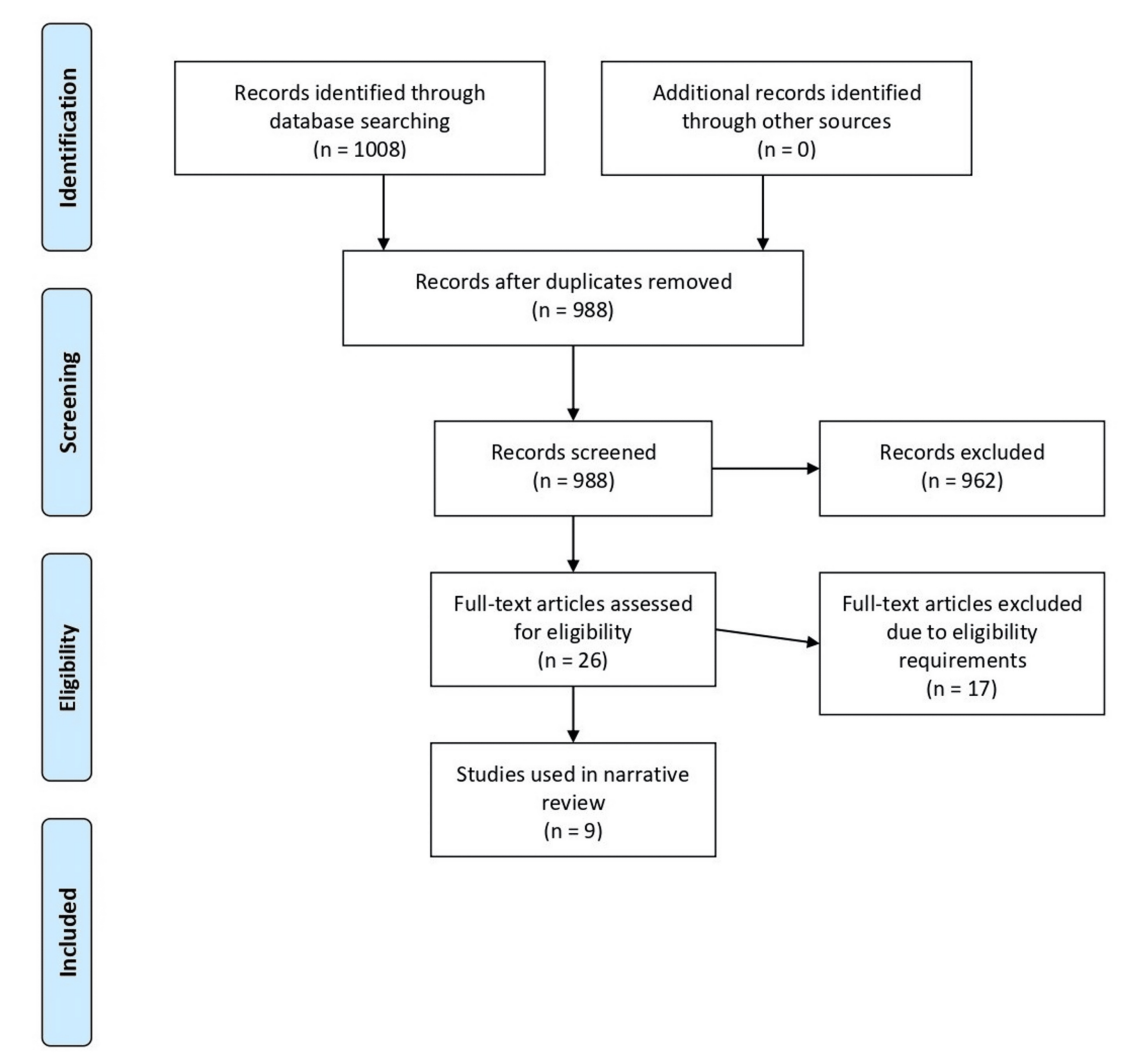
PRISMA flow diagram PRISMA, Preferred Reporting Items for Systematic reviews and Meta-Analyses Source: [18]

Out of the 1,008 articles identified through our comprehensive search, records were exported to Rayyan, an online systematic review tool used to manage search results, remove duplicates, and streamline the screening of titles and abstracts, ultimately yielding 26 relevant articles for inclusion. Four authors carried out this process jointly. After carefully reviewing the full texts, we identified nine studies that met our eligibility criteria. Data were extracted from these studies, and challenges, opportunities, and future directions were grouped into common themes. The process is shown in the Preferred Reporting Items for Systematic reviews and Meta-Analyses (PRISMA) flowchart (Figure 1) [18].

Results

A literature search of databases identified 1,008 studies, as shown in Figure 1. After duplicates were removed, 988 studies remained, of which 962 were excluded based on title and abstract screening. Following a full-text review of the remaining 26 papers, only nine studies fully met the eligibility criteria according to the previously described inclusion and exclusion guidelines. Studies that met the inclusion criteria are summarized in Table 2.

**Table 2:** Summary of study characteristics OHS, open-heart surgery

Author and date	Title of article	Country/region	Center(s) where the study was conducted	Key challenges highlighted
Eze and Ezemba (2007) [1]	Open-heart surgery in Nigeria: indications and challenges	Nigeria	Various historical national-level data, including the University of Nigeria Teaching Hospital, Enugu, and other early centers	Limited number of procedures, inadequate infrastructure, lack of trained staff, insufficient funding, and low public awareness
Nwiloh et al. (2018) [2]	Challenges to providing open heart surgery for 186 million Nigerians	Nigeria	National-level/countrywide analysis	Health system limitations, inadequate funding, workforce shortages, poor access, and reliance on foreign centers
Falase et al. (2016) [3]	The challenges of cardiothoracic surgery practice in Nigeria: a 12 years institutional experience	Nigeria	Lagos State University Teaching Hospital	Minimal OHS volume, insufficient equipment, lack of outcome data, funding constraints, workforce and infrastructure gaps
Falase et al. (2013) [9]	Open heart surgery in Nigeria; a work in progress	Nigeria	Lagos State University Teaching Hospital, Ikeja, Lagos	Low case volume, limited skilled workforce, high perioperative mortality, and inadequate local capacity
Alioke et al. (2024) [11]	Navigating the challenges in setting up a sustainable open-heart surgery unit in a resource-constrained environment in Northern Nigeria: model and strategies	Nigeria	Federal Medical Centre, Abuja (FMC Abuja), Northern Nigeria	Resource constraints, limited technology, dependence on visiting teams, and workforce training gaps
Osinaike (2016) [14]	Open-heart surgery programme in Nigeria: the good, the bad, and the ugly	Nigeria	Nationwide/country-level	Economic downturns and funding instability; frequent resource shortages; infrastructural decay; inadequate and irregular maintenance of equipment; lack of coordinated national planning; and dependence on foreign collaborations and missions for sustainability
Nwiloh et al. (2012) [19]	Cardiac surgical experience in northern Nigeria	Nigeria	National Hospital, Abuja, and Ahmadu Bello University Teaching Hospital, Zaria	Limited local experience, follow-up challenges, anticoagulation management issues, and dependence on visiting teams
Salami et al. (2018) [20]	Early results of open-heart surgery for acquired heart diseases in Ibadan, Nigeria	Nigeria	University College Hospital, Ibadan	Funding constraints, reliance on visiting international teams, limited infrastructure, postoperative anticoagulation management, small patient volumes, late patient presentation, and program sustainability dependent on building a fully independent local team
Onakpoya et al. (2017) [21]	Early experience with open heart surgery in a pioneer private hospital in West Africa: the Biket Medical Centre experience	Nigeria	Biket Medical Centre, Osogbo, Osun State (private hospital)	While private sector OHS proved feasible, sustainability is threatened by reliance on visiting external teams and foreign missions for expertise, consumables, and support; challenges in establishing durable local infrastructure (ICU capacity, supply chains, and consistent access to consumables); long-term follow-up and system-wide sustainability remain uncertain

Challenges 

Financing and affordability: The high cost of OHS remains one of the greatest barriers to accessing cardiac care in Nigeria [1,9,22]. Although several centers have achieved technical success, affordability continues to determine who receives surgery. In a recent study from Enugu, 96% of patients paid out of pocket, with costs ranging from $5,076 to $7,614, a burden described as “catastrophic” for almost all families [22]. Similarly, Falase et al. (2013) [9] reported direct procedure costs ranging from $6,230 for atrial septal defect closure to $11,200 for mitral valve replacement, underscoring the financial inaccessibility for the average Nigerian [9].

Historical data show that this challenge is not new. Eze and Ezemba (2007) estimated that OHS without a prosthesis typically costs $4,800, and with a prosthesis $5,600, with ICU costs of $85 per night and Rocephin at $20 per gram [1]. They found that patients relied primarily on household funding (65.5%), while government support accounted for only 9%. At the time, Nigeria’s minimum wage was $44-62 per month; in 2025, it stands at ₦70,000 (~$47/month), indicating that affordability has barely improved in two decades.

Other reports have echoed these concerns. Osinaike and Nwiloh et al. noted that the lack of government subsidy, high cost of imported consumables, and minimal insurance coverage make programs financially unsustainable [14,23]. Reviews also emphasize that limited funding and the absence of cardiac care within the national insurance package continue to exclude most Nigerians from lifesaving surgery [1,3,22].

Overall, the cost of OHS in Nigeria remains prohibitively high relative to income, and without deliberate government financing or inclusion under national insurance, equitable access will remain out of reach for the majority.

Shortage of workforce: A major challenge to sustaining OHS in Nigeria is the chronic scarcity of trained OHS personnel across cadres. Even long-established programs in centers such as Lagos and Enugu face difficulty maintaining full teams of surgeons, perfusionists, anesthetists, and ICU nurses, contributing to intermittent surgical activity and continued reliance on visiting mission teams to perform complex procedures, visits that are often too brief and infrequent to allow for regular skills transfer [1,9,19,22,24].

The workforce shortage is compounded by inadequate local training opportunities for new cardiac staff, with few institutions offering structured cardiothoracic programs. Many trainees experience low procedural exposure and insufficient mentorship, which reduces confidence and discourages specialization in the field [1,14,25]. Brain drain and poor retention strategies further exacerbate this challenge: many cardiac-team personnel report dissatisfaction with remuneration and infrastructure, leaving programs vulnerable when individual staff migrate to better-resourced settings [2].

Inadequate infrastructure: OHS involves capital-intensive equipment, which is often lacking in most African countries. In Nigeria, where the availability of a simple diagnostic tool like transthoracic echocardiography is a luxury in many hospitals, the unavailability of necessary technological equipment significantly impedes the growth of OHS [23]. This technological limitation affects the entire clinical process, from diagnosis to surgery [23].

These procedures also require stable infrastructure, such as a reliable power supply, and facilities like 24-hour laboratory support, an active blood bank, and cardiac catheterization support, which are rarely available in Nigeria, as noted by Falase et al. [9]. With an unreliable power supply, hospitals rely heavily on generators, which often break down due to constant use. There is also a significant shortage of catheterization facilities, and laboratory support is often limited or unavailable after working hours [9,14]. Two studies highlighted limited blood bank services as a factor affecting OHS services [9,19]. These represent some of the minimum standards required to operate a facility offering OHS.

The overarching reason for infrastructure and equipment inadequacy identified in most studies relates to funding: associated importation costs [1], irregular funding for equipment maintenance [9], and lack of government support [14].

Low volume of cases: While the shortage of skilled workforce is one of the greatest challenges facing OHS in Nigeria, it is further perpetuated by the low volume of cardiac surgical cases performed in the country [1]. Nwiloh et al. [2] reported that approximately 100 heart surgeries are performed annually in Nigeria. For a country with a population of over 180 million people, this volume falls well below the World Health Organization target projection of 72,000 cases per annum and the Pan African Society of Cardiothoracic Surgery target of 7,200 cases per annum [2].

Practice makes perfect. This is especially true in surgical training, where proficiency is achieved and maintained through repetitive practice of surgical skills. Unfortunately, the current low case volume hinders the acquisition, maintenance, and mastery of essential cardiac surgical technical skills [2]. It limits hands-on exposure and leads to the de-skilling of already trained staff [9].

A temporary solution often employed is sending surgeons abroad for “refresher courses.” However, the benefit of this measure is usually short-lived, as surgeons lose newly acquired skills shortly after returning home due to minimal practice caused by the low case volume [2].

Another concerning effect of low case volume is its indirect impact on public confidence in the abilities of local cardiothoracic surgeons. Medical tourism is predominant, especially for open-heart surgeries. This contributes to the low case volume while creating a vicious cycle: local surgeons have fewer opportunities to achieve positive outcomes, which in turn limits public confidence and reduces local referrals [9,14].

It is important to note that low case volume is not an independent issue but a result of multiple factors. One contributing factor is the inability of patients to afford the procedure. In a country where healthcare is largely paid out of pocket, this greatly impacts the number of cases performed each year [2].

Poor incentives and retention: The lack of incentives has also affected the growth of cardiac surgery in Nigeria. This has contributed to a “brain-drain syndrome,” resulting in poor retention of skilled medical professionals [1]. Several reasons underlie this trend.

Many practicing cardiothoracic surgeons have raised concerns that their remuneration is not proportional to their years of training, especially when compared with other colleagues. This situation is exacerbated by the lack of private practice opportunities to supplement income [2].

Another reason cited by professionals is frustration over limited career progression and lack of opportunities to use and maintain cardiac surgical skills [2]. Without adequate incentives, these factors, and others, have driven surgeons to seek opportunities in other countries.

Systems and governance: The progress of OHS in Nigeria mirrors the broader systemic challenges of the national health system. Systemic and governance failures are among the most persistent obstacles to scaling sustainable OHS in the country. Despite the presence of skilled personnel and historical milestones, such as the first successful OHS at the UNTH in 1974, progress has been slow. Multiple institutional reports describe a weak national framework for cardiac surgery in terms of planning, funding, training, and regulation, resulting in centers operating largely independently with inconsistent leadership, unstable funding, and limited accountability structures [1,3]. Program fragility is manifested in irregular theater lists, reliance on visiting mission teams, and difficulties maintaining equipment and consumables, problems that interrupt service continuity and impede skill consolidation [9,23,24].

A key challenge within the governance structure is the poor referral system among hospitals. Some institutions reportedly delay or avoid referring patients to centers where surgery is available, often due to professional rivalry or institutional protectionism. Osinaike [14] described such tendencies as detrimental to patient outcomes and to the collective advancement of OHS in the country. This fragmented approach prevents optimal case flow and denies many patients timely, lifesaving surgery.

Further systemic challenges include weak administrative processes, inconsistent equipment maintenance, and the absence of centralized registries to track patient outcomes and surgical capacity [9,23]. Limited policy support and reliance on out-of-pocket payments further constrain institutional planning and sustainability, leaving many programs vulnerable to interruptions [22].

Strengthening governance through a national cardiovascular surgery program, regional centers of excellence, and strategic public-private partnerships (PPPs) would enhance accountability, promote standardization, and ensure equitable access across the country [11,14].

Opportunities

International partnerships: Across the Nigerian literature, international collaboration and visiting cardiac surgical missions are repeatedly identified as a cornerstone for sustaining OHS activity [3,9,11]. Several programs started with external support from foreign teams, which helped address gaps in local expertise, equipment, and surgical volume, while providing an operational framework for emerging centers [1,9]. These missions offered Nigerian surgeons, perfusionists, and nursing staff valuable practical exposure to cardiopulmonary bypass, perioperative care, and more complex procedures, although the extent of hands-on experience and follow-up training varied across programs [2,14]. More recent experiences highlight that when mission support is coordinated with local team development and clear educational objectives, it can foster sustainable capacity building and progressively reduce reliance on visiting teams [11].

The Nigerian experience demonstrates that international partnerships yield the greatest benefit when integrated into a broader local capacity-building agenda. Reports emphasize that models combining visiting missions with structured training, mentorship, and gradual transfer of responsibility to local staff, supported by investment in infrastructure and equipment maintenance, tend to achieve more durable outcomes [2,20]. Aligning such collaborations with national policy and consistent funding can transform them from episodic service efforts into sustainable training platforms that strengthen Nigeria’s OHS capacity.

Hybrid model: The private sector has begun to play a growing complementary role in Nigeria’s OHS landscape. Experience from Biket Medical Centre in Osogbo illustrates that, with proper planning and collaboration, cardiac surgery can be safely performed in non-governmental hospitals, achieving encouraging short-term outcomes despite resource limitations [21]. Such centers benefit from leaner administrative structures, faster procurement processes, and greater operational autonomy, allowing them to innovate around logistics and financing.

Hybrid models that blend public institutional frameworks with private investment and mission support are increasingly viewed as a pragmatic pathway toward sustainable cardiac surgery growth in Nigeria. Reports show that while most early OHS programs were government-driven, recent progress has stemmed from partnerships uniting public infrastructure with private or philanthropic resources, facilitating improved equipment maintenance, staffing continuity, and gradual capacity transfer [2,9,14]. These arrangements often include shared clinical governance, cost-recovery mechanisms, and local staff training during visiting missions, creating a platform that blends service delivery with capacity building.

The gradual expansion of Nigeria’s OHS ecosystem through private and hybrid initiatives presents a pragmatic path toward long-term self-reliance. Evidence from recent reports shows that diversifying funding streams, strengthening management systems, and building stable workshops can help bridge the gap between service demand and capacity. When anchored in coherent national policy and appropriate regulatory oversight, these evolving models could transform OHS from an episodic, mission-driven service into a continuous and sustainable national enterprise [11,19,20].

Growing local demand: Nigeria is witnessing an increasing burden of cardiovascular diseases, creating an urgent epidemiologic need for sustainable OHS services. Once considered rare, congenital and acquired heart diseases, such as rheumatic valve disorders and ischemic heart disease, are now being diagnosed more frequently due to improved awareness and diagnostic access [1,2,9]. Early institutional reports reveal that the majority of surgical indications in Nigeria include valvular heart disease, atrial septal defects, and tetralogy of Fallot, conditions that are both prevalent and surgically correctable [1].

The demand for OHS is also being driven by demographic transitions. As Nigeria’s population ages and urbanization accelerates, lifestyle-related cardiovascular morbidity is rising, further expanding the candidate pool for surgical intervention [2,9]. However, current local capacity addresses only a fraction of the population, leading many patients to seek expensive care abroad or forgo treatment entirely [3,14,22].

Despite systemic challenges, emerging data from both public and private centers suggest a strong willingness among patients to pursue surgery locally when services are available [21]. In Enugu, most patients financed surgery through personal savings or family support, highlighting both the financial burden and the determination to access care [22]. Similarly, early experience from a pioneering private hospital demonstrated that even in resource-limited settings, locally delivered OHS is feasible and well-accepted [21].

The growing epidemiologic need, combined with demonstrated patient demand, presents a major opportunity for scaling OHS in Nigeria. Strengthening surgical programs will not only reduce outbound medical tourism but also ensure timely intervention for life-threatening cardiac diseases [1,3,9,11,14,22,23]. Strategic investment in cardiac units, supported by health insurance expansion and public-private collaboration, could bridge the widening gap between need and access.

Way Forward 

The future of OHS in Nigeria depends on robust government policies that guarantee sustainable financing and equitable access to cardiac care. Currently, most patients still pay out of pocket for surgery, and only a small proportion are covered by insurance, underscoring the urgent need for a comprehensive national insurance scheme to enhance financial protection and service utilization [22,26,27]. Recent expert commentary has therefore called on the government to integrate cardiac surgery into the NHIA coverage to promote access and equity [28]. Health policies should consequently be reformed to ensure that OHS and other high-cost procedures are covered by the NHIA, while fostering PPPs to improve funding, training, and facilities nationwide.

Targeted government funding, take-off grants, and performance-based subsidies are also essential to establish and sustain cardiac centers. In addition, charitable foundations and non-governmental organizations play key roles in bridging resource gaps. However, the attraction of foreign agencies has been weakened by political instability and episodes of violence, highlighting the need for a stable and secure environment [1].

Several authors have advocated moving away from short-term, mission-based models due to sustainability concerns, whereas others recommend continued supervision by visiting teams until adequate skill transfer occurs [9,23]. Sustainable progress will therefore rely on strategic collaboration between local and international teams, emphasizing mentorship, technology exchange, and long-term partnerships [9,23,29]. Indeed, some centers have adopted PPP models in which private entities contribute to program development and share profits [14], while others have resumed surgery under PPP arrangements with foreign teams that facilitate skill transfer and infrastructure growth [20].

Finally, a strong workforce, effective governance, and infrastructure investment, supported by training, incentives, and coordinated planning, are indispensable for achieving a self-sustaining, resilient national OHS program [1,9,11,23,25,29,30].

## Conclusions

OHS in Nigeria stands at a pivotal crossroads. While progress has been made, sustainability remains elusive. Persistent challenges, such as inadequate funding, a shortage of trained personnel, and dependence on foreign missions, continue to limit consistent service delivery. Yet, success stories from centers that have adopted structured training, strategic partnerships, and public-private collaboration demonstrate that local capacity can indeed thrive.

Moving forward, government-led policy reform, integration of cardiac surgery into national health insurance, and investment in workforce development are imperative. With coordinated action and long-term commitment, Nigeria can build a self-sustaining, accessible, and resilient cardiac surgery program capable of meeting the needs of its growing population.

## References

[1] Eze JC, Ezemba N (2007). Open-heart surgery in Nigeria: indications and challenges. Tex Heart Inst J.

[2] Nwiloh J, Smit F, Mestres C (2018). Challenges to providing open heart surgery for 186 million Nigerians. Niger J Cardiovasc Thorac Surg.

[3] Falase B, Sanusi M, Animasahun A (2016). The challenges of cardiothoracic surgery practice in Nigeria: a 12 years institutional experience. Cardiovasc Diagn Ther.

[4] OGUNYALE K, Mac-Leva F, Mac-Leva KO (2024). Budget: citizens allocated N524 monthly for healthcare. https://www.icirnigeria.org/budget-citizens-allocated-n524-monthly-for-healthcare/?tztc=1.

[5] Awoyemi BO, Makanju AA, Mpapalika J, Ekpeyo RS (2023). A time series analysis of government expenditure and health outcomes in Nigeria. J Public Health Afr.

[6] The Abuja Declaration: Ten Years On. https://iris.who.int/items/862622b2-6147-49c0-b5d1-0409b64df759.

[7] Adewole DA (2024). The National Health Insurance Authority of Nigeria and implications for universal health coverage. Ann Ib Postgrad Med.

[8] Effiong FB, Dine RD, Hassan IA, Olawuyi DA, Isong IK, Adewole DA (2025). Coverage and predictors of enrollment in the state-supported health insurance schemes in Nigeria: a quantitative multi-site study. BMC Public Health.

[9] Falase B, Sanusi M, Majekodunmi A, Animasahun B, Ajose I, Idowu A, Oke A (2013). Open heart surgery in Nigeria; a work in progress. J Cardiothorac Surg.

[10] Nwafor IA, Vickram A, Osenmobor KO (2020). Surgical 'safari' vs. educational program: experience with international cardiac surgery missions in Nigeria. Braz J Cardiovasc Surg.

[11] Alioke II, Idoko FL, Abiodun OO (2024). Navigating the challenges in setting up a sustainable open-heart surgery unit in a resource-constrained environment in northern Nigeria: model and strategies. Brazil J Cardiovasc Surg.

[12] Nigeria Open-Heart Surgery Registry. https://www.nigeriaheartregistry.com.

[13] Lindsay Gandolfo Bringing life saving heart surgery to children in Nigeria. Columbia Surgery.

[14] Osinaike BB (2016). Open-heart surgery programme in Nigeria: the good, the bad and the ugly. Niger Postgrad Med J.

[15] Dixon‐Woods M, Fitzpatrick R, Roberts K (2001). Including qualitative research in systematic reviews: opportunities and problems. J Eval Clin Pract.

[16] Sandelowski M, Docherty S, Emden C (1997). Qualitative metasynthesis: issues and techniques. Res Nurs Health.

[17] Collins JA, Fauser BC (2005). Balancing the strengths of systematic and narrative reviews. Hum Reprod Update.

[18] Moher D, Liberati A, Tetzlaff J, Altman DG (2009). Preferred Reporting Items for Systematic Reviews and Meta-Analyses: the PRISMA statement. PLoS Med.

[19] Nwiloh J, Edaigbini S, Danbauchi S, Babaniyi I, Aminu M, Adamu Y, Oyati A (2012). Cardiac surgical experience in northern Nigeria. Cardiovasc J Afr.

[20] Salami M, Akinyemi O, Adegboye V (2018). Early results of open-heart surgery for acquired heart diseases in Ibadan, Nigeria. Niger J Cardio.

[21] Onakpoya UU, Adenle AD, Adenekan AT (2017). Early experience with open heart surgery in a pioneer private hospital in West Africa: the Biket medical centre experience. Pan Afr Med J.

[22] Ujunwa FA, Chinawa JM, Arodiwe I (2021). Health-care financing among patients admitted for open-heart surgery in Enugu. Niger J Med.

[23] Nwiloh JO, Oludara MA, Adebola PA. (2014). Heart surgery practice in Sub Saharan Africa: single Nigerian institutional midterm results and challenges. World J.

[24] Oludara MA, Nwiloh J, Fabamwo A, Adebola P (2014). Commencing open heart surgery in resource limited countries: lessons from the LASUTH experience. Pan Afr Med J.

[25] Okoli C, Nwanna-Nzewunwa O, Onyinyechukwu Adaeze A, Etukokwu E, Okoli C (2024). The barriers to cardiothoracic surgery training in Nigeria: key insights from trainees. Cureus.

[26] Eze OI, Iseolorunkanmi A, Adeloye D. (2024). The National Health Insurance Scheme (NHIS) in Nigeria: current issues and implementation challenges. J Glob Health Econ Policy.

[27] Alawode GO, Adewole DA (2021). Assessment of the design and implementation challenges of the National Health Insurance Scheme in Nigeria: a qualitative study among sub-national level actors, healthcare and insurance providers. BMC Public Health.

[28] Biodun Busari (2024). Nigeria has only 80 heart surgeons - Association President, Etiuma. https://punchng.com/nigeria-has-only-80-heart-surgeons-association-president-etiuma/#google_vignette.

[29] Nwafor IA, Eze JC, Nwafor MN (2019). Establishing a sustainable open heart surgery programme in Nigeria, a low-income country: which is the best model?. Cardiol Young.

[30] Okonta KE, Tobin-West CI (2016). Challenges with the establishment of congenital cardiac surgery centers in Nigeria: survey of cardiothoracic surgeons and residents. J Surg Res.

